# Exploiting genomic surveillance to map the spatio-temporal dispersal of SARS-CoV-2 spike mutations in Belgium across 2020

**DOI:** 10.1038/s41598-021-97667-9

**Published:** 2021-09-17

**Authors:** Nena Bollen, Maria Artesi, Keith Durkin, Samuel L. Hong, Barney Potter, Bouchra Boujemla, Bert Vanmechelen, Joan Martí-Carreras, Tony Wawina-Bokalanga, Cécile Meex, Sébastien Bontems, Marie-Pierre Hayette, Emmanuel André, Piet Maes, Vincent Bours, Guy Baele, Simon Dellicour

**Affiliations:** 1 Laboratory of Clinical and Epidemiological Virology, Department of Microbiology, Immunology and Transplantation, Rega Institute for Medical Research, KU Leuven, Leuven, Belgium; 2 Department of Human Genetics, CHU Liège, and Medical Genomics, GIGA Research Center, University of Liège, Liège, Belgium; 3 Department of Clinical Microbiology, University of Liège, 4000 Liège, Belgium; 4 Spatial Epidemiology Lab (SpELL), Université Libre de Bruxelles, CP160/12, 50, av. FD Roosevelt, 1050 Bruxelles, Belgium

**Keywords:** Phylogenetics, Phylogeny

## Abstract

At the end of 2020, several new variants of SARS-CoV-2—designated variants of concern—were detected and quickly suspected to be associated with a higher transmissibility and possible escape of vaccine-induced immunity. In Belgium, this discovery has motivated the initiation of a more ambitious genomic surveillance program, which is drastically increasing the number of SARS-CoV-2 genomes to analyse for monitoring the circulation of viral lineages and variants of concern. In order to efficiently analyse the massive collection of genomic data that are the result of such increased sequencing efforts, streamlined analytical strategies are crucial. In this study, we illustrate how to efficiently map the spatio-temporal dispersal of target mutations at a regional level. As a proof of concept, we focus on the Belgian province of Liège that has been consistently sampled throughout 2020, but was also one of the main epicenters of the second European epidemic wave. Specifically, we employ a recently developed phylogeographic workflow to infer the regional dispersal history of viral lineages associated with three specific mutations on the spike protein (S98F, A222V and S477N) and to quantify their relative importance through time. Our analytical pipeline enables analysing large data sets and has the potential to be quickly applied and updated to track target mutations in space and time throughout the course of an epidemic.

## Introduction

Coronavirus disease 2019 (COVID-19) is caused by the severe acute respiratory syndrome coronavirus 2 (SARS-CoV-2) that has been responsible for over 117 million positive cases and 2.6 million deaths at the time of writing (March 9, 2021). While real-time sequencing efforts have for instance been applied in recent epidemics of Ebola^1^ and Lassa virus^2^, the COVID-19 pandemic marks the first time where whole-genome sequencing capacity has been widely available to the public health sector from its emergence. In this context, it is thus worth considering the role that phylogenetic and phylogeographic analyses can fulfil as a surveillance tool and what information can be gleaned from them when deployed during an active epidemic^3^. Genomic surveillance can also allow detecting new mutations which could affect the transmissibility or pathogenicity of the virus, or keep track of mutations that may alter or impair current diagnostic tools, putative drug interaction or epitope exposition, which may affect the efficacy of vaccination efforts.

On December 14, 2020, the 202012/01 SARS-CoV-2 variant of concern (viral lineage B.1.1.7) was discovered in the United Kingdom (UK) and is now strongly suspected to be associated with an increased transmissibility, with an effective reproduction number increased by a value ranging from 0.4 to 0.7 (Volz et al. 2020, Imperial College Report 42). Furthermore, two other SARS-CoV-2 variants discovered in late 2020 (N501.V2, viral lineage B.1.351, discovered in South Africa, and N501.V3, viral lineage P.1, discovered in Brazil) have also been suspected to be associated with a higher capacity of human-to-human transmission^4,5^.

In the fall of 2020, Belgium was hit with a second wave of infections that dwarfed the first (with up to 700 daily new hospitalisations at its peak). International media took note of the severity, with one article even citing Liège as the “epicenter” of the European second wave (https://abcnews.go.com). Equally alarming was the elevated per capita mortality rate of COVID-19 in Belgium, which ranks among the highest in the world at 172 deaths per 10,000 people (https://coronavirus.jhu.edu/data/mortality). Even if it has been recognised that the critical circulation of the virus in nursing homes has played an important role^6^, the cause of this high mortality rate is not yet fully understood. The nature of the Belgian epidemic, in combination with the recent discovery in the UK, South Africa and Brazil of new SARS-CoV-2 variants of concern, motivated Belgian authorities and the scientific community to launch the implementation of a more ambitious genomic surveillance program. During 2020, genomic surveillance efforts in Belgium have been mainly carried by molecular sequencing at the Universities of Leuven and Liège^7^, but will as of 2021 involve a broader nationwide network of laboratories and research teams to increase the weekly sequencing capacity. With the goal to sequence over 1000 SARS-CoV-2 genomes per week, but also to perform retrospective sequencing to attain an improved coverage across the entire country, the resulting massive collection of viral genomic sequences requires a rapid but reliable analytical procedure to monitor the circulation of variants and/or mutations of concern.

In a previous study, we established an analytical workflow to deal with these issues at the country level^8^. This workflow can be summarised in three steps: first, a time-calibrated maximum-likelihood (ML) tree is inferred. In a second step, the aim is to identify introduction events into the geographic area of interest. For this purpose, the ML tree is used as an empirical tree in a Bayesian discrete trait analysis, which annotates the nodes according to their inferred location. Finally, the third step consists in running a continuous phylogeographic analysis along each clade corresponding to a distinct introduction event identified in the previous step. This way, we perform a fine-grained analysis of the dispersal history of viral lineages in the geographic area of interest. A step-by-step guide to this workflow as well as all necessary code can be found in the GitHub repository associated with this project (https://github.com/sdellicour/sars-cov-2_liege). As a proof of concept, we here focus on the analysis of Belgian sequences collected between March and November 2020 in the province of Liège, Belgium. This decision is motivated by the particularly dense and relatively time-homogeneous sampling of SARS-CoV-2 genomes available during that time period for the province of Liège, as well as by the fact that this province was particularly impacted by the second COVID-19 epidemic wave in Belgium, which reflects an important circulation of the virus during that period and the likely co-occurrence of various viral variants. Specifically, we aim to illustrate how to efficiently map the spatio-temporal dispersal of key mutations of interest at a regional level. Unravelling such spatio-temporal distributions is of key importance when monitoring specific mutations or viral variants associated with a potential impact on the epidemiological dynamics of the disease^9^. Our study also showcases the importance of novel analytical approaches and pipelines—to avoid that phylogenetic tools constitute a bottleneck in the time-constrained setting that the ongoing pandemic has researchers working in^10^—to track the evolution and spread of mutations of interest in increasingly large genomic data sets on a daily basis.

## Materials and methods

### SARS-CoV-2 sequencing

**Figure 1:**
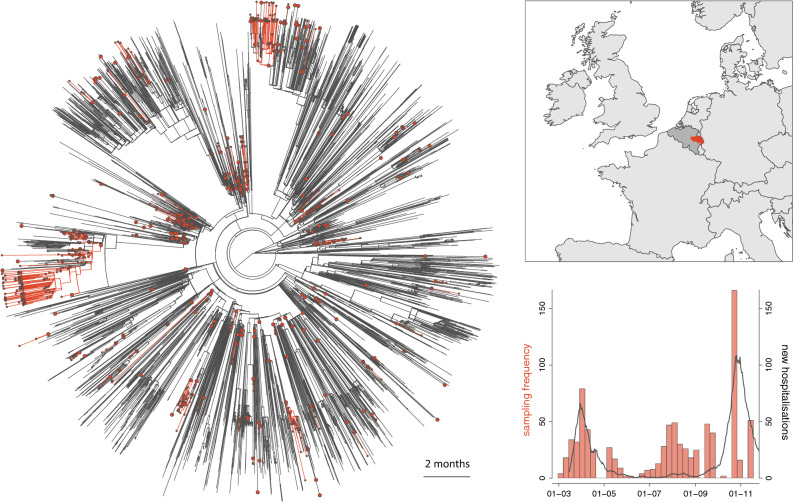
Time-scaled phylogenetic tree in which we identified phylogenetic clades introduced in the province of Liège (Belgium). We delineated those clades by performing a discrete phylogeographic reconstruction along the time-scaled phylogenetic tree while only considering two potential ancestral locations: “province of Liège” and “other location”. We identified a minimum of 244 lineage introductions (95% HPD interval = [239–250]) for 689 sequences sampled in the province of Liège. On the phylogeny, lineages circulating in the province of Liège are highlighted in red, and red nodes correspond to the most ancestral node of each clade. Apart from the phylogenetic tree, we also report a map of western Europe highlighting Belgium (in darker grey) and the province of Liège (in red), as well as a graph displaying both the number of sequences sampled through time and the daily number of new hospitalisations in the province of Liège (source: national public health institute of Belgium, Sciensano, https://epistat.wiv-isp.be/covid).

Between March 1, 2020, and November 30, 2020, we sequenced a total of 869 SARS-CoV-2 genomes from clinical samples collected across the province of Liège, Belgium (Fig. 1). We extracted RNA from clinical samples (300 µl) via a Maxwell 48 device using the Maxwell RSC Viral TNA kit (Promega) with a viral inactivation step using Proteinase K, following the manufacturer’s instructions. RNA elution occurred in 50 µl of RNAse free water. Reverse transcription was carried out via SuperScript IV VILOTM Master Mix, and 3.3 l of the eluted RNA was combined with 1.2 l of master mix and 1.5 l of H_2_O. This was incubated at 25 °C for 10 min, 50 °C for 10 min and 85 °C for 5 min. PCR used Q5® High-Fidelity DNA Polymerase (NEB), the primers and conditions followed the recommendations in the sequencing protocol of the ARTIC Network (https://artic.network/ncov-2019). More recent samples used the 1200 bp amplicons described by Freed et al.^11^. We multiplexed the samples following the manufacturer's recommendations using the Oxford Nanopore Native Barcoding Expansion kits 1–12, 13–24, and 96 in conjunction with Ligation Sequencing Kit 109 (Oxford Nanopore). We carried out sequencing on a Minion using R9.4.1 flow cells.

### Inference of a time-scaled phylogenetic tree

We followed the analytical workflow previously described by Dellicour et al., which enables efficient phylogeographic analysis of the dispersal history of SARS-CoV-2 lineages across a given study area^8^. The first main step of this workflow consisted in inferring a time-scaled phylogenetic tree. This approach allows us to significantly reduce the computation time needed to obtain results, as constructing a maximum-likelihood phylogenetic tree inference is less time-consuming than performing a Bayesian one. For this purpose, we first retrieved all non-Belgian sequences present in the European Nextstrain^12^ build on December 1, 2020. Nextstrain uses a subsampling scheme in order to construct a real-time snapshot tree of the larger SARS-CoV-2 epidemic and phylogenetic diversity. Sequences are divided into groups organised by location, sublocation, year and month. Each group is then sampled equally (when possible) in order to construct a data set of a predetermined size. We also selected all available Belgian SARS-CoV-2 genomic sequences available on that day (for a total of 5657 sequences, with 2114 sequences sampled in Belgium and 869 sequences sampled in the province of Liège). After having gathered all accession numbers, we downloaded the latest whole genome alignment from GISAID (www.gisaid.org). At the time, 495 Belgian sequences were yet to be uploaded to GISAID, so we added them to the multiple sequence alignment using MAFFT 7.453^13^. We subsequently cleaned the alignment by trimming the 5′ and 3′ untranslated regions and gap-only sites. To obtain a maximum-likelihood phylogeny, we ran IQ-TREE 2.0.3^14^ under a general time-reversible (GTR) model^15^ of nucleotide substitution with empirical base frequencies and four free site rate categories^16^, which was selected as the optimal model using IQ-TREE’s ModelFinder tool. We also discarded one sequence that failed IQ-TREE’s sequence composition test. After having discarded this outlier, we time-calibrated the phylogeny using TreeTime 0.7.4^17^. To replicate the Nextstrain workflow as closely as possible, we specified a clock rate of 8 × 10^–4^ in TreeTime and set to filter samples that deviated more than four interquartile ranges from the root-to-tip regression^8^.

### Preliminary discrete phylogeographic inference

The second main step of the workflow consisted of performing a preliminary phylogeographic analysis using the discrete diffusion model^18^ implemented in the software package BEAST 1.10^19^. The objective of this second step was to identify independent introduction events of SARS-CoV-2 lineages into the province of Liège. To this end, we used the time-scaled phylogenetic tree (obtained in the previous step) as a fixed empirical tree and only considered two possible ancestral locations: “province of Liège” and “other location”, as described in Dellicour et al.^8^. Bayesian inference through Markov chain Monte Carlo (MCMC) was performed on this empirical tree for 3 × 10^5^ iterations and sampled every 1,000 iterations. MCMC convergence and mixing properties were inspected using the program Tracer 1.7^20^ to ensure that effective sample size (ESS) values associated with estimated parameters were all > 200. After having discarded 10% of sampled trees as burn-in, a maximum clade credibility (MCC) tree was generated using TreeAnnotator 1.10^19^. We used the resulting MCC tree to delineate phylogenetic clades corresponding to independent introduction events into the province of Liège, i.e. clades whose most likely location of origin was the province of Liège according to the discrete diffusion model. In practice we compared the locations assigned to each pair of nodes connected by the phylogenetic branches of this MCC tree, i.e. the most probable location inferred at internal nodes and the sampling location for tip nodes to identify such introduction events. We considered an introduction event to be the case when the location assigned to a node was “province of Liège” and the location assigned to its parent node in the tree was “other location”^8^.

### Continuous and post hoc phylogeographic analyses

The third main step of the workflow consisted of performing a spatially-explicit phylogeographic inference with the relaxed random walk (RRW) diffusion model^21^ implemented in BEAST 1.10^19^, using a Cauchy distribution to model the among-branch heterogeneity in diffusion velocity. This way, we performed a distinct continuous phylogeographic reconstruction for each clade occurring in the province of Liège and identified by the previous discrete phylogeographic inference as clustering at least three sequences with a known municipality of origin, again fixing a time-scaled subtree as an empirical tree^8^. Given that the RRW diffusion model does not accept identical coordinates for different sequences, we retrieved sampling coordinates from a point randomly selected within the municipality of origin for each sampled sequence. Each Markov chain was run for 5 × 10^8^ generations and sampled every 10^5^ generations. As with the discrete phylogeographic inference, MCMC convergence/mixing properties were assessed with Tracer, and MCC trees (one per clade) were obtained with TreeAnnotator after discarding 10% of sampled trees as burn-in. We then used functions available in the R package “seraphim”^22,23^ to extract spatio-temporal information embedded within the same 1000 posterior trees and visualise the continuous phylogeographic reconstructions. As motivated above, we specifically focused on phylogenetic branches corresponding to lineage dispersal events occurring within the province of Liège (Fig. 1).

### Tracking specific mutations in the analysis of viral lineages

In addition to the three main steps detailed above, we used Nextclade v.0.10.1 (https://www.npmjs.com/package/@neherlab/nextclade) to identify all amino acid mutations relative to the Wuhan-Hu-1/2019 reference genome (MN908947.3), and build on our spatially-explicit phylogeographic reconstruction to map clades of the tree associated with specific mutations in the spike protein. In practice, we considered that the common ancestor of sampled sequences that all carry a specific mutation was likely to carry it too. In other words, if all terminal nodes associated with an internal node carry a particular mutation, then this internal node was tagged with said mutation. In particular, we targeted three specific mutations in the spike protein (S98F, A222V, and S477N) due to their rapid expansion and dissemination across Europe during the summer months^9^.

## Results and discussion

**Figure 2:**
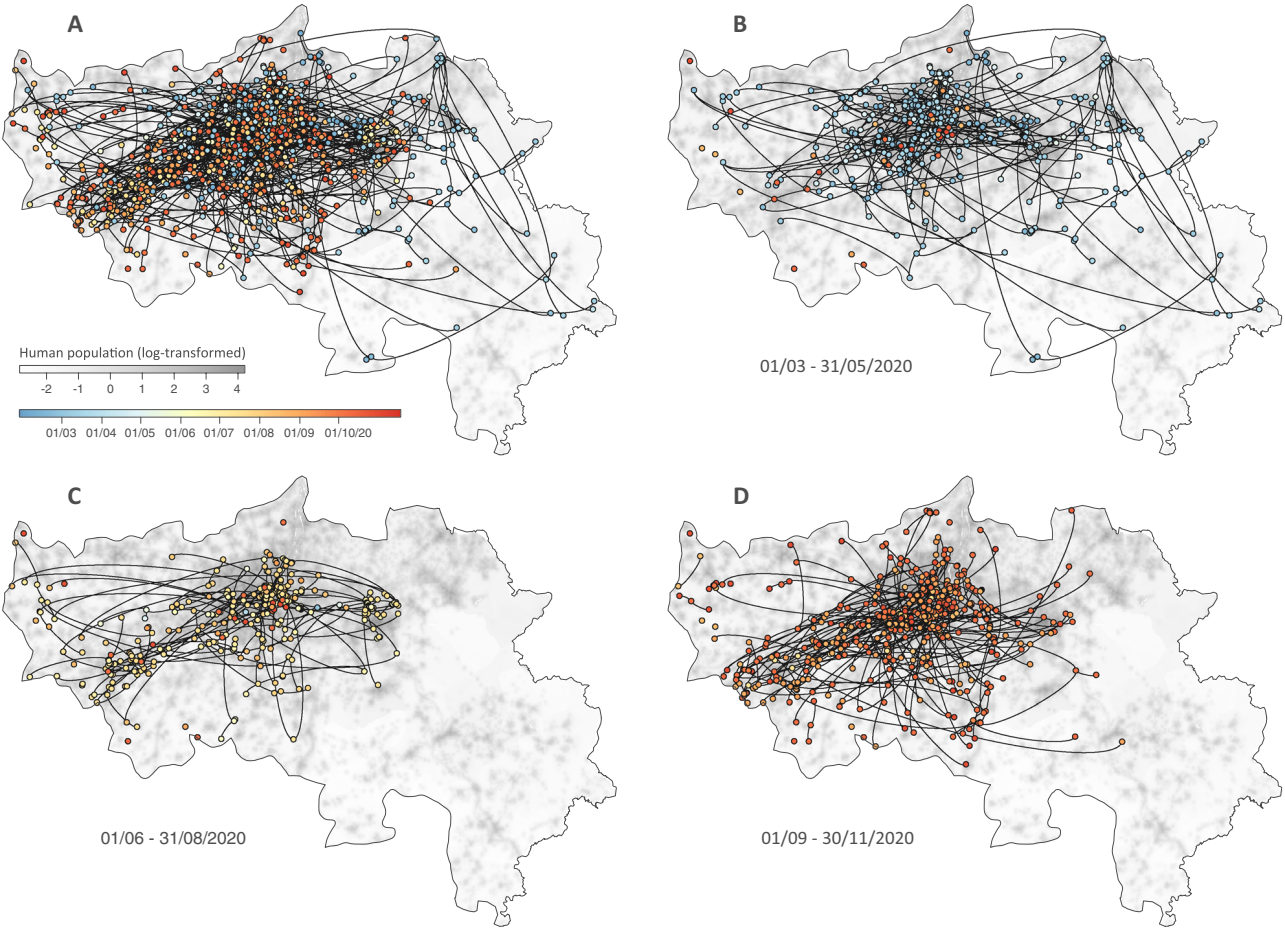
Spatially-explicit phylogeographic reconstruction of the dispersal history of SARS-CoV-2 lineages in the province of Liège. Continuous phylogeographic inference was performed along each clade originating in the province of Liège identified by the initial discrete phylogeographic analysis. For each clade, we mapped all maximum clade credibility (MCC) branches located in the province of Liège (**A**), as well as subsets of phylogenetic branches whose terminal nodes occurred during three specific time periods: from March 1 to May 31, 2020 (corresponding to the first epidemic wave; **B**), from June 1 to August 31, 2020 (corresponding to the summer period; **C**), and from September 1 to November 30, 2020 (corresponding to the second epidemic wave; **D**). Dispersal directions of viral lineages are indicated by the edge curvature (dispersal direction is anti-clockwise) and phylogenetic nodes are coloured according to their time of occurrence.

In total, we identified a minimum of 244 lineage introductions into the province of Liège (95% HPD interval = [239–250]) for a total of 689 sequences sampled across the region (Fig. 1). This illustrates the relative importance of introduction events to settle local transmission chains within this relatively restricted study area. Spatially-explicit phylogeographic reconstructions along resulting SARS-CoV-2 clades circulating in the province of Liège illustrate and confirm that lineage dispersal events occurred across the study area during all phases of the epidemic (Fig. 2), i.e. during the first (March–May, 2020) and second (September–November, 2020) epidemic waves as well as during the summer period (June–August, 2020).

**Figure 3:**
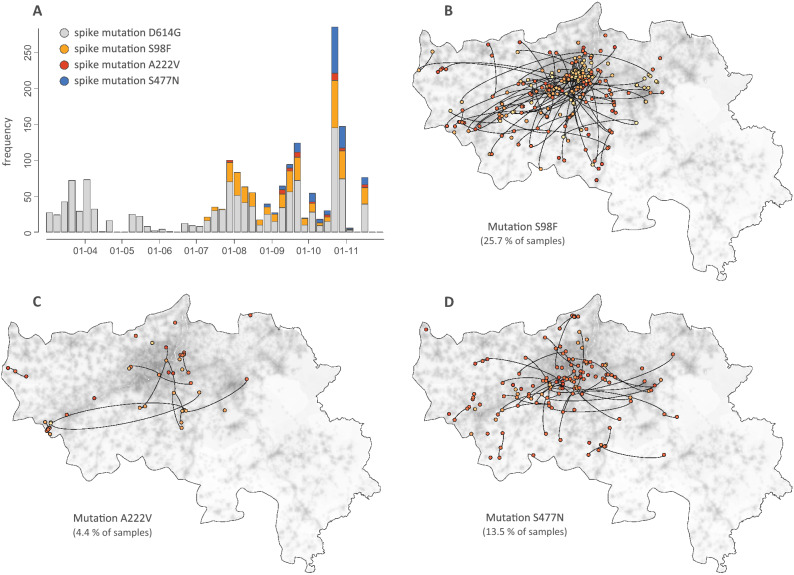
Spatially-explicit phylogeographic reconstruction of the dispersal history of SARS-CoV-2 lineages associated with specific spike protein mutations in the province of Liège. We report the estimated temporal evolution of the frequency of the main spike mutations in the province of Liège (**A**), as well as the cartography of phylogenetic nodes and branches associated with the target mutations S98F (**B**), A222V (**C**), and S477N (**D**). Similar to Fig. 1, phylogenetic branches are superimposed on a map of population density (log-transformed) and phylogenetic nodes are coloured according to their time of occurrence using the same colour scale used in Fig. 2.

We subsequently used spatially-explicit phylogeographic reconstructions to track the dispersal of specific spike protein mutations across the study area (Fig. 3). Among the three target mutations, S98F has been detected with the highest frequency (in 25.7% of our samples). This mutation started to circulate in July and was either introduced from unsampled locations where it was previously undetected or, more likely, derived from regional/national circulating lineages. In contrast, the A222V mutation has been detected far less in the study area (in only 4.4% of our samples), despite notably higher frequencies of detection at the end of the summer in surrounding countries^9^. Having emerged in early summer in north-eastern Spain, the variant carrying this mutation has shown an impressive colonisation across the continent, most likely boosted by more permissive travelling schedules during summer in most European countries. In the province of Liège however, it has been detected several times but does not seem related to notable regional circulations, which contrasts with European regions such as England, where it has been established as one of the most frequent spike mutations identified in samples. Finally, the S477N spike mutation was first detected at the end of the summer and displays moderate regional circulation and detection frequency (13.5%). It has recently been reported that S477N might strengthen the binding of the SARS-COV-2 spike with the human ACE2 receptor^24^, which could potentially be associated with enhanced viral transmissibility. While the other target mutations are currently not suspected to be associated with different transmissibility (or pathogenicity), it is now more important than ever to keep monitoring the spatio-temporal diversity of mutations to anticipate the potential need to track variants of concern that could impact the dynamic of the epidemic or diagnose evasion during and after the vaccination campaigns.

Following in the footsteps of other recently developed approaches^25,26^, our previously-developed analytical pipeline focuses on computational efficiency while compromising as little as possible on analytical rigor^8^. As such, phylogenetic inference is performed using maximum likelihood instead of Bayesian inference, which would not be feasible on large data sets. However, by still performing Bayesian phylogeographic inference later on in the workflow, we accommodate uncertainty in the ancestral location reconstruction and the exact sampling locations that are protected by privacy law. At this time, it is not possible to introduce new sequences into an already existing phylogeny. Instead, the entire pipeline would have to be run again when new data becomes available. In future work, we will evaluate the applicability and feasibility of novel approaches to accommodate phylogenetic uncertainty through parsimonious phylogenetic placement initiatives^27,28^, which may be able to provide a measure of uncertainty for newly sequenced genomes placed onto an existing phylogeny that was obtained in a previous analysis (i.e. an approach that has been shown to improve analysis time requirements^28^). Another limitation in phylogeographic analyses is the possible effect of sampling bias. Hence, any conclusions have to be carefully interpreted and situated against the sampling pattern and sampling effort, although recent work in the field have tried to mitigate the effects of sampling bias^29,30^.

Our current pipeline follows official guidelines set by the WHO, which has advised countries to increase routine and systematic sequencing surveillance of SARS-CoV-2 samples in order to monitor the emergence and circulation of SARS-CoV-2 variants. Genomic surveillance efforts have already proven to not have been in vain, as new mutations have been detected in a number of countries. Three such instances, i.e. the aforementioned 202012/01 VOC (also known as lineage B.1.1.7) discovered in the UK, the 501.V2 variant (also known as lineage B.1.351) in South Africa and the 501.V3 variant (also known as lineage P.1) in Brazil—which have acquired multiple mutations that differentiate them from previously dominant strains—are currently of concern and require close monitoring. Given its recent jump from animals, it was not unexpected for the transmissibility of SARS-CoV-2 to rise as it adapts to human hosts^31^. Variants with higher transmissibility are by definition not guaranteed to follow the same dispersal dynamics as their predecessor. Tracking the relative frequency of these variants as well as analysing their dispersal dynamics are crucial in order to implement adapted prevention and control strategies. In order to achieve these goals, we believe the analytical pipeline presented in this study is of interest as it can rapidly be applied to obtain a spatio-temporal picture of the circulation and diversity of viral lineages.

## Data Availability

R scripts and related files needed to run all the analyses are all available at https://github.com/sdellicour/sars-cov-2_liege.
